# Editorial: Recent advances in quality control technology for fresh fruits and vegetables

**DOI:** 10.3389/fnut.2025.1697510

**Published:** 2025-12-12

**Authors:** Li Wang, José Pinela, Lin Chen, Dan Chen

**Affiliations:** 1 Key Laboratory of Agricultural Product Fine Processing and Resource Utilization, Ministry of Agriculture and Rural Affairs, Anhui Engineering Research Center for High Value Utilization of Characteristic Agricultural Products, School of Food and Nutrition, Joint Research Center for Food Nutrition and Health of IHM, Anhui Agricultural University, Hefei, China; 2 National Institute for Agricultural and Veterinary Research (INIAV, I.P.), Vila do Conde, Portugal; 3 CIMO, LA SusTEC, Instituto Politécnico de Bragança, Campus de Santa Apolónia, Bragança, Portugal; 4 School of Chemistry, Chemical Engineering and Biotechnology, Nanyang Technological University, Singapore, Singapore; 5 College of Food Science and Engineering, Yangzhou University, Yangzhou, China

**Keywords:** postharvest preservation, fermentation, enzymatic browning, shelf life extension, food processing technologies, digestive health, FODMAPs, functional foods

Fresh fruits and vegetables are essential components of a healthy diet due to their rich content of vitamins, minerals, dietary fiber, and bioactive compounds (1). However, their high perishability presents significant challenges in preserving nutritional and sensory properties during storage and distribution. Addressing these challenges requires a deeper understanding of postharvest physiology and innovative processing strategies that minimize nutrient degradation and reduce food losses. Recent advances in physical, chemical, and biological technologies are opening new avenues to extend shelf life, enhance digestibility, and improve the functional value of plant-based foods. The studies in this Research Topic contribute to this growing body of knowledge, offering mechanistic insights and practical approaches to improve food processing, reduce waste, and meet the needs of health-conscious consumers and sustainable food systems.

The study by Alowo et al. investigated the effects of thermal and non-thermal processing methods (specifically roasting and malting) on the content of fermentable oligo-di-monosaccharides and polyols (FODMAPs) in commonly consumed varieties of millet, sorghum, soybeans, and sesame. The authors found that crop variety plays a significant role in determining FODMAP levels. Notably, elite varieties such as Maksoy 3N (soybean), Seremi 2 (millet), and Narosorg 2 (sorghum) exhibited significantly lower levels of oligosaccharides and excess fructose compared to indigenous varieties, with percentage reductions ranging from 10.2% to 73.9%. These findings underscore the importance of varietal selection in balancing nutritional quality and digestive health. In terms of processing, malting proved to be more effective than roasting in reducing total FODMAP content, particularly excess fructose, consistently lowering it to within the recommended threshold of < 0.15 g/100 g across all grain types. Moreover, malting reduced total oligosaccharides and total FODMAPs in soybean and sesame by more than 50%. However, despite its relative effectiveness, malting alone did not achieve the recommended thresholds of < 0.3 g/100 g for oligosaccharides and < 0.5 g/100 g for total FODMAPs, which define suitability for low-FODMAP diets. The results highlight the potential of combining crop improvement with optimized processing techniques to develop more digestible grain-based foods, particularly for individuals managing gastrointestinal disorders such as irritable bowel syndrome.

Expanding on the theme of food preservation, the study by Wang et al. examined the synergistic effects of co-treatment with 1-methylcyclopropene (1-MCP) and hydrogen sulfide (H_2_S) on the postharvest quality of strawberries, with a specific focus on sugar and energy metabolism during cold storage. Using a combined application of 1.0 μL/L 1-MCP and 0.8 mmol/L sodium hydrosulfide (a donor of H_2_S), the authors demonstrated that this dual treatment was significantly more effective than either compound alone in maintaining postharvest quality. The co-treatment effectively suppressed decay progression, reduced weight loss, and preserved fruit firmness and visual appeal over 15 days of storage at 4 °C. Mechanistically, these benefits were associated with regulation of sugar metabolism: the treatment inhibited sucrose-degrading enzymes such as acid invertase (AI) and neutral invertase (NI), while promoting the activities of sucrose synthase (SS) and sucrose phosphate synthase (SPS), resulting in higher sucrose content and reduced levels of glucose and fructose. Additionally, the combined treatment enhanced energy metabolism by upregulating key enzymes in energy metabolism, including succinate dehydrogenase (SDH), cytochrome c oxidase (CCO), H^+^-adenosine triphosphatase (ATPase), and Ca^2+^-ATPase, leading to increased ATP levels and improved cellular energy charge. These metabolic adjustments contributed to the maintenance of cell structural integrity and overall fruit quality. These findings highlight the promising potential of 1-MCP and H_2_S co-treatment as an effective strategy to extend shelf life and preserve the nutritional and sensory attributes of strawberries. Furthermore, this approach offers a strong scientific basis for broader postharvest applications across a range of fruits and vegetables.

Fermentation emerges as another powerful tool in the study by Dong et al., which explored the impact of *Lactobacillus acidophilus* fermentation on the processing and digestive characteristics of honeysuckle polyphenols, aiming to enhance the functional value of honeysuckle-based beverages. Using optimized fermentation conditions (35 °C for 19 h with a 3% inoculation rate), the authors found that fermentation significantly improved both antioxidant capacity and α-glucosidase inhibitory activity after simulated gastrointestinal digestion. Importantly, the bioavailability of key bioactive compounds was enhanced in the fermented beverage: total phenolics, flavonoids, and chlorogenic acid showed relative increases of 35%, 26%, and 44%, respectively, compared to the unfermented control. The fermented beverage also maintained a relatively stable pH during cold storage at 4 °C, despite a moderate decline in viable bacterial count, color, and sensory score. Nevertheless, the functional integrity of the beverage was preserved, suggesting strong potential for the development of probiotic and antioxidant-rich honeysuckle products. Overall, the findings provide a solid scientific foundation for leveraging fermentation to improve both the health benefits and commercial appeal of plant-based functional beverages.

In the area of enzymatic browning of fresh-cut fruit and vegetables, the opinion article by Cao et al. offers a fresh perspective by emphasizing the underappreciated yet pivotal role of phenylalanine ammonia-lyase (PAL). While most research focuses on polyphenol oxidase (PPO) and peroxidase (POD) as primary contributors to browning, the authors present compelling evidence that PAL is equally critical. Acting upstream in the phenylpropanoid pathway, PAL drives the synthesis of phenolic substrates and pigmented compounds. Mechanical wounding from fresh-cut processing strongly induces PAL gene expression and enzyme activity in various crops, including lettuce, apples, taro, and peaches. This activation results in both increased biosynthesis of colored flavonoids and higher levels of phenolics available for oxidation by PPO or POD. Challenging the traditional view that browning stems mainly from PPO activity, the authors argue that PAL may serve as a key initiating factor by regulating precursor accumulation. Supporting this, chemical inhibition of PAL significantly reduces browning in multiple fresh-cut products. Furthermore, the lack of consistent correlation between PPO or POD activity and browning intensity in some crops highlights the relevance of PAL-mediated pathways. The authors propose targeting PAL activity as a promising, yet underexplored, strategy for browning control and advocate further research into its molecular regulation, including gene identification and the development of inhibitors or genetic tools. By shifting focus upstream, this work provides a theoretical foundation for more effective methods to preserve the appearance, shelf life, and nutritional value of minimally processed fruits and vegetables.

Taken together, these studies highlight the multifaceted progress in food science, where innovations in processing, preservation, and biochemical understanding converge to address critical challenges. From reducing FODMAPs to support digestive health, to employing synergistic treatments to extend shelf life, harnessing fermentation for functional enhancement, and redefining the control of enzymatic browning, these insights collectively chart a promising path toward healthier, more sustainable, and higher-quality plant-based food products. As the field continues to evolve, integrating such diverse strategies will be essential for meeting the growing demands of global food systems and increasingly discerning consumers, driving innovation that enhances not only nutritional and functional value, but also sensory appeal and overall food experience.
